# Experimentally Determined Long Intrinsically Disordered Protein Regions Are Now Abundant in the Protein Data Bank

**DOI:** 10.3390/ijms21124496

**Published:** 2020-06-24

**Authors:** Alexander Miguel Monzon, Marco Necci, Federica Quaglia, Ian Walsh, Giuseppe Zanotti, Damiano Piovesan, Silvio C. E. Tosatto

**Affiliations:** 1 Department of Biomedical Sciences, University of Padua, 35131 Padua, Italy; alexander.monzon@unipd.it (A.M.M.); rvrmarco@gmail.com (M.N.); federica.quaglia8@gmail.com (F.Q.); giuseppe.zanotti@unipd.it (G.Z.); 2 Bioprocessing Technology Institute, A*STAR, Singapore 138668, Singapore; curly.walsh@gmail.com

**Keywords:** intrinsically disordered proteins, protein flexibility, structure missing residues, disordered regions

## Abstract

Intrinsically disordered protein regions are commonly defined from missing electron density in X-ray structures. Experimental evidence for long disorder regions (LDRs) of at least 30 residues was so far limited to manually curated proteins. Here, we describe a comprehensive and large-scale analysis of experimental LDRs for 3133 unique proteins, demonstrating an increasing coverage of intrinsic disorder in the Protein Data Bank (PDB) in the last decade. The results suggest that long missing residue regions are a good quality source to annotate intrinsically disordered regions and perform functional analysis in large data sets. The consensus approach used to define LDRs allows to evaluate context dependent disorder and provide a common definition at the protein level.

## 1. Introduction

Intrinsically disordered proteins (IDPs) and regions (IDRs) defy the classical structure-function paradigm [1,2,3]. However, IDPs/IDRs classification is still quite ambiguous due to their remarkable versatility [2]. As a result, various flavors of the disorder have been proposed, some based on amino acid composition [4], flexibility [5,6], functional roles coupled with conservation [7], and the ability of many disordered proteins to bind specifically to other proteins by folding upon binding events [8,9]. Perhaps the simplest distinction is between proteins with short and long disordered regions. While short disordered regions in the Protein Data Bank (PDB) are usually associated with flexible linkers or loops in folded proteins [10], long disordered regions (LDRs) are special, since they seem to behave differently in function [2,10,11,12] and evolution [13]. Furthermore, LDRs fall under the definition of intrinsically disordered domains, which are known to be involved in protein-protein recognition thanks to their structural and functional independence from the rest of the protein [14].

Those regions which are poorly defined in the electron density map and have consequently poorly defined atomic coordinates are informed as missing residues. Missing residues in protein structures have been widely used as a proxy to identify IDPs/IDRs [15,16,17]. Nowadays, the PDB [18], the major repository of three-dimensional structures for proteins and nucleic acids, has more than 150,000 structures. PDB is mainly composed of X-ray (89%) and nuclear magnetic resonance (NMR) (8%) structures, with a small number of Cryogenic electron microscopy (cryo-EM) (ca. 3%) and other techniques. A large-scale analysis is possible by using high-quality experimental data from thousands of protein structures. Structurally, the disorder can range from regions that in solution are totally flexible to those that present two or more different but defined conformations [19]. Unfortunately, these two cases are often difficult to distinguish in an X-ray crystal structure, particularly at low or medium resolution. In fact, in structures with resolutions higher than 2.5 Å it is possible to observe loops or short areas crystallized in different conformations. However, at lower resolutions, these flexible regions are not visible in the electron density map. Consequently, the corresponding residues are left out of the molecular model. Cryo-EM also provides an ever-increasing number of relatively high-resolution structures (with the exception of very few cases at a resolution better than 2 Å, the vast majority of them are at best 3 Å) deposited in PDB [20]. Contrary to X-ray diffraction, cryo-EM structures allow, at least partially, to distinguish the presence of LDRs from conformationally flexible segments [21].

In this manuscript, those residues that are missing from the polypeptide chain (despite being present in the primary structure) are defined as “disordered”, without attempting to distinguish between disordered, flexible or mobile regions. Only LDRs of at least 30 consecutive residues are considered in order to disregard missing residues that may occur due to low resolution or experimental conditions and to capture functional disordered regions [14,22]. As different structures of the same protein may contain varying amounts of disorder, two consensus approaches were used to define unequivocally LDRs.

## 2. Results

### 2.1. Quality of Structures with Disordered Regions

One possible factor contributing to the presence of missing residues could be the quality of the crystallographic structures. The structure quality estimation for NMR and cryo-EM models is difficult to assess in order to be comparable with crystallographic structures. Additionally, the way that cryo-EM determines the resolution is different [23], and consequently, their maps and models could not be equivalent [24]. For the above-mentioned reasons and the small contribution of these methods to our dataset (2% of the structures), we only considered X-ray structures to perform the consensus disorder definition. 

**Figure 1:**
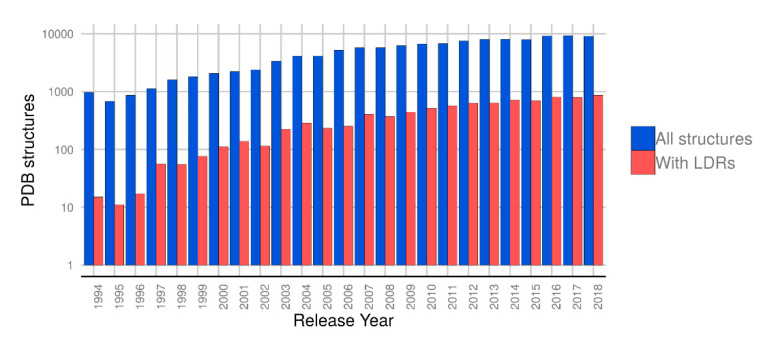
Growth of proteins with Long Disorder Regions (LDRs) in the PDB. In blue, the amount of structures deposited by year in the PDB. In red, the amount of deposited structures with at least one LDR. The y-axis is expressed in logarithmic scale.

Looking at the release dates for the PDB structures in our dataset (Figure 1), it can be observed that most structures with LDRs have been deposited in the last five years, suggesting that the improvements of crystallization techniques are allowing the growth of crystals of partially disordered or flexible proteins [25,26]. While the amount of structures deposited remains roughly stable in the last years, a steady increment of PDB files with LDRs can be seen.

**Figure 2:**
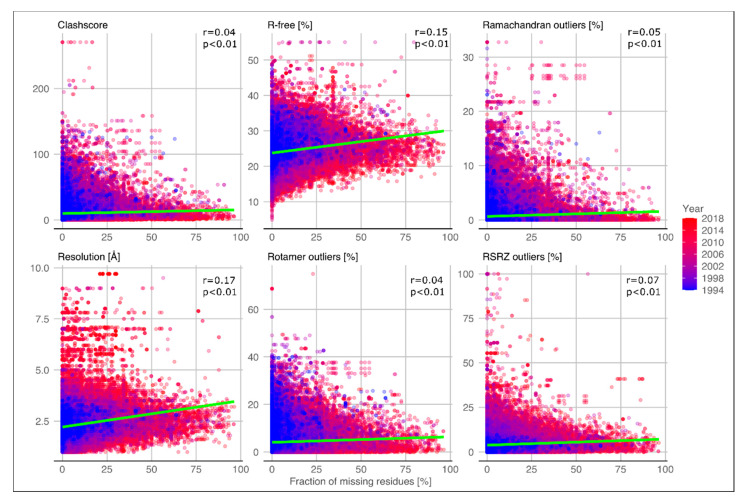
Scatter plots of different structure quality metrics and fraction of missing residues. On X-axis the fraction of missing residues calculated for the PDB chain (SEQRES). On Y-axes the different quality metrics corresponding specified on the title of each subplot. The color represents the year of deposition in the protein data bank (PDB). The green line represents the linear regression. Pearson’s correlation coefficients (r) and P-values (p) are shown for each subplot.

In addition, PDB structures with LDRs seem to have the same overall quality as those without. In a previous work, structure validation reports were introduced in the PDB and a set of metrics are used to assess structure quality [27]. Other recent studies demonstrated that structure quality has been improved in the last ten years, suggesting that each new deposited structure will be better than the average quality in the PDB [25,26]. In Figure 2 the same set of metrics, namely resolution, R-free, clashscore, Ramachandran outliers, rotamer outliers, and RSRZ outliers (normalized real-space R-factor), are shown in relation to the disorder content (fraction of missing residues). Pearson’s correlation is almost zero for all metrics and only moderately positive for resolution and R-free parameters (0.17 and 0.15 respectively). Moreover, the same set of measures were correlated with the LDR length for each PDB and chain (Figure S1). Similar to the analysis shown in Figure 2, Pearson’s correlation coefficients are also close to zero for most of the metrics. Low correlations might suggest that LDRs are not a mere experimental artifact but rather represent a structural-functional feature.

### 2.2. Distribution of Disordered Regions

**Figure 3:**
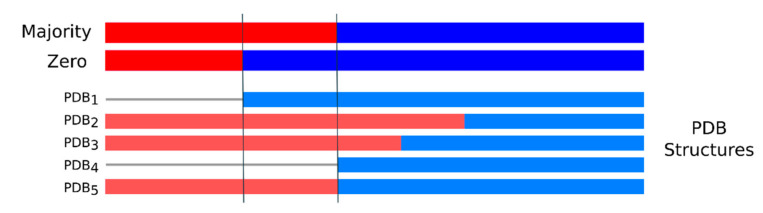
Intrinsically disordered region (IDR) definition at the protein level. Example of the majority and zero consensus definitions when more than one structure maps to the same protein sequence. Missing residue regions are in red, structured parts in blue and unknown residues as a gray line. The N-terminus is always disordered with an IDR according to the zero consensus. PDB_1_ limits the length of the IDR in the zero consensus because it is the only one which has structured residues in the region between the black lines. Consequently, the IDR of the majority consensus is longer and contains those residues which are missing in at least 3 out of 5 PDB chains (more than 50%). Unknown residues, i.e., those not considered to grow the crystal, are excluded and do not affect the consensus definitions.

When evaluating disorder at the protein level multiple PDB structures can provide alternative observations which can be combined in multiple ways to generate different types of consensus. Figure 3 shows an example of the differences between the two consensus approaches adopted, a permissive “majority” and a conservative “zero” consensus (see Section 4.1). In total, the majority rule provides 3133 proteins with at least one LDR, where 2758 (88%) of them are confirmed with the zero consensus. Another 1123 proteins have a single PDB entry and coincide in the two consensus definitions (see Table 1 for dataset composition). Other proteins do not have missing regions (structured proteins) or have only short disordered regions between 5 and 30 residues (short disordered proteins). These three sets of proteins have on average the same fraction of structured residues (ca. 60%–63%). The disorder content of the LDR dataset is 14.1% and rises to 19.2% when unknown residues, i.e., those not considered to grow the crystal, are filtered out. About half of the total missing residues are found inside long regions and a large fraction of residues is not defined (35% “Unknown”, Table 1). The fraction of unknown residues could harbor a further source of LDRs resisting crystallization.

**Figure 4:**
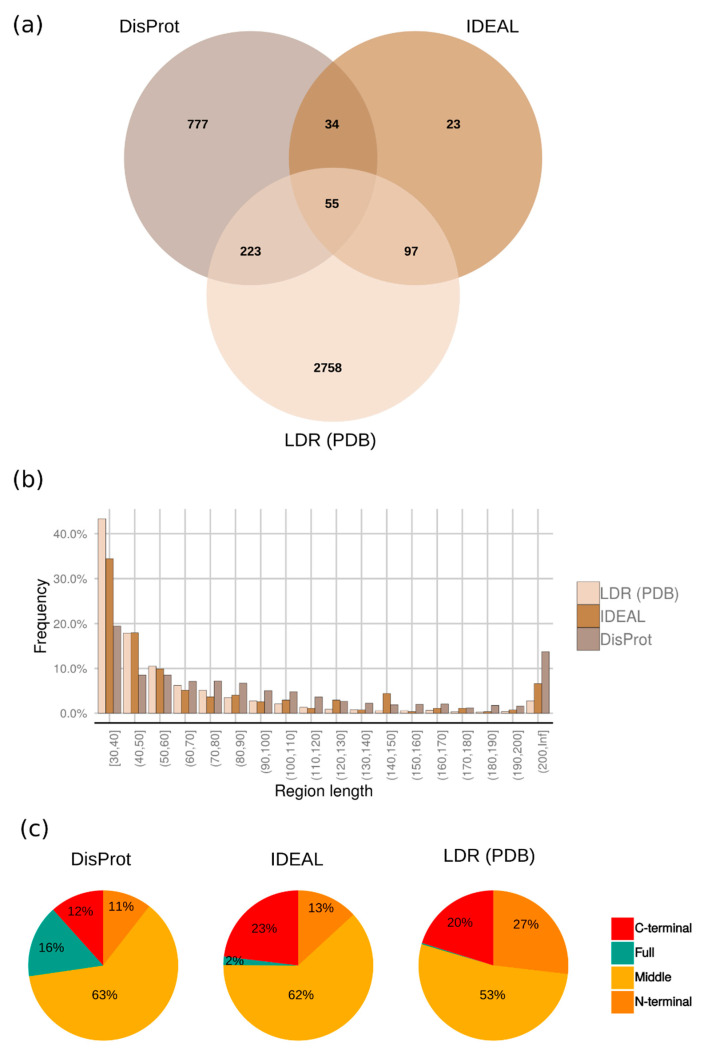
Comparison with Long Disordered Regions (LDRs) from DisProt and IDEAL. (**a**) Venn diagram showing the overlap between our LDR dataset from PDB, DisProt, and IDEAL. Only proteins with LDR regions (at least 30 residues) were considered for IDEAL and DisProt databases. (**b**) Length distribution of the long-disordered regions found in DisProt, IDEAL and LDR (PDB) dataset. The data is grouped by bins of ten residues. (**c**) Fraction of LDRs which fall in the N- or C-terminus, middle of the protein or cover the entire sequence (full). Tails (N- and C-) refers to LDRs at the ends (20% of the total residues) of the full protein length.

The number of proteins with LDR obtained from this analysis is larger than in the manually curated databases, DisProt [28] (1089 proteins), and IDEAL [29] (209 proteins). Only a small fraction of proteins are shared between our dataset and IDEAL (152 proteins), while DisProt has a larger overlap (278 proteins) (Figure 4a). Our data does not replace IDEAL and DisProt, which have put a big effort to manually curate IDPs/IDRs and their function from literature. However, it offers a much larger and complementary experimental LDR source. DisProt and IDEAL consider different experimental techniques to annotate IDPs/IDRs, this allows on one hand a more thorough evaluation of LDRs boundaries and on the other hand a more sensitive detection of those LDRs that could escape single techniques. DisProt also shows a good fraction of fully disordered proteins or extremely long disordered regions (longer than 200 residues) annotated from alternative biochemical techniques, e.g., circular dichroism, sensitivity to proteolisis, etc. [28,30].

The distribution of LDR length is shown in Figure 4b, along with DisProt [28] and IDEAL [29] databases for comparison, showing an exponential decay with increasing length. The decrease is consistent with IDEAL and DisProt. 50% of the regions in our dataset are between 30–44 amino acids, however, DisProt presents a bigger amount of extremely long regions (at least 200 residues) compared to IDEAL and the LDR set. Our dataset has 93 proteins with these extreme LDRs which represent a real niche of the PDB. Furthermore, inspecting LDRs length obtained by the zero consensus, we did not find any statistically significant difference with LDRs in the majority consensus.

Although each protein may contain more than one LDR, one region is the norm (2773 proteins, 88.5%), with two being somewhat common (315 proteins, 10%). Most of LDRs are present in the middle of the proteins in DisProt, IDEAL and LDR set, followed by C-terminal and N-terminal (Figure 4c).

The amino acid composition of LDRs in our dataset follows the characteristic compositional bias of IDPs/IDRs, it is enriched in charged and hydrophilic amino acids and depleted in hydrophobic ones [31]. In Figure S2 we showed the fold increase (or decrease) using as background the distribution of amino acids in the PDB (SEQRES sequences) for LDRs in the majority and zero consensus. We also calculated the enrichment for all structured (observed) residues in the PDB and manually curated disordered regions in DisProt. The LDRs amino acid composition is similar to DisProt. The zero consensus is even more similar to DisProt since it is more conservative.

### 2.3. Function of Proteins with LDRs

While some of the LDRs may be the result of poor diffraction quality, it is well established now that the majority of them have functional roles [1,2,3,28,32]. To further support this statement, we manually curated 99 regions corresponding to 93 proteins with unusual LDRs by using the same curation procedure that we adopt for DisProt [28] (Table S1). 77% of those LDRs have literature evidence showing that they are disordered or unstructured, while 23% are never mentioned in the scanned literature. Probably, missing residues were added during the structure refinement process for those proteins for which we do not have any clue about the disorder, or simply the authors were not interested in characterizing or mentioning the disordered region. Interestingly, even some of the largest LDRs in X-ray structures are likely functional disordered regions instead of a result of specific or accidental experimental conditions, yielding a high-quality dataset. The majority of proteins of this subset are now available in DisProt release 2020_05.

The size of the dataset allows us to perform function enrichments. We performed a Gene Ontology (GO) [33,34] enrichment analysis to analyze the functional role of proteins with LDR and fully disordered proteins. This kind of analysis is useful to inspect the range of functions that could have proteins composed of structured and disordered domains. There is still missing a specific ontology to perform function assignment to singular protein regions.

**Figure 5:**
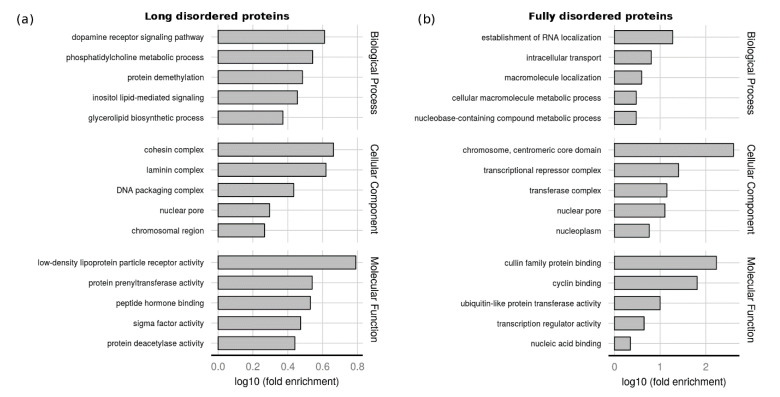
GO-terms enrichment analysis. Five most enriched GO-terms in the three ontologies for (**a**) long disordered proteins and (**b**) Fully disordered proteins. The background set was all UniProt sequences with at least one PDB structure. The x-axis shows the logarithmic increase compared to the background (see Methods for details).

The background used was all proteins with at least one PDB structure. The median disorder content in the LDR dataset is 15%, so we considered fully disordered proteins with more than 70% of disordered residues. These proteins are well studied and represent a specific functional class. The five most enriched terms in each ontology are shown in Figure 5. Intrinsically disordered regions (IDRs) function has been extensively studied in literature, not only in particular cases [2,35] but also in large scale studies [36,37]. The function of proteins with LDR differs from fully disorder proteins. The LDR set (Figure 5a) is enriched in molecular function terms, commonly associated with the IDPs/IDRs activity. The terms low-density lipoprotein particle binding and peptide hormone binding are related to the ability of IDRs to bind small molecules, macromolecules, or other proteins. Protein prenyltransferase activity and protein deacetylase activity terms refer to the role of IDRs as effectors, interacting and modifying other proteins activities [2], while sigma factor activity is connected with the IDPs involved in transcription regulation. In biological process ontology, the LDR set is enriched in signal transduction terms (inositol lipid-mediated signal transduction and dopamine receptor signaling pathway), protein demethylation, biosynthesis, and in the phosphatidylcholine metabolism. Among cellular components, proteins with LDRs are mostly present in the nucleosome, chromosomes, and protein-containing complexes. This last term is the ancestor of three (cohesin, laminin, and DNA packaging complexes) out five of the most enriched terms and can be associated with the capability of IDPs/IDRs to interact with different partners. Fully disordered proteins (Figure 5b) are associated with developmental processes, nucleic acid binding, and transcription regulation, and are mainly present in the cell nucleus. This suggests a different functional role in the cell supporting a recent study based on disorder predictions [36]. In summary, many GO terms previously associated with disorder have been confirmed by our analysis and support the reliability of our LDR set [2,11,12,38].

### 2.4. Assessment of Disorder Predictors

Information about missing electron densities has traditionally been used to train disorder predictors. In Table 2 and Table S2 we summarized the evaluation of MobiDB predictions against the majority and zero LDR datasets, respectively. Despite some methods tend to reach good precision (best precision 0.87, MobiDB-lite), all have a lower sensitivity (best recall 0.65, VSL2b) indicating that a large fraction of LDR remains undetected. The two datasets seem not to differ significantly, in the “zero” LDR dataset ESpritz-X reaches a slightly better MCC (0.461 “zero” and 0.456 “majority”). As shown in a previous work [10], ESpritz-X reaches the best MCC (0.454) also against the DisProt dataset. Although the performance appears similar to the LDR dataset, in the DisProt dataset predictors have a higher sensitivity and lower specificity. This may be explained by the fact that the DisProt dataset is more balanced (higher disorder content). In another work [39] predictors performance was evaluated against the same type of dataset, i.e., considering missing residues from the PDB. Predictors performance was slightly better. However, in that work LDRs were defined as longer than 20 residues instead of 30, indicating that shorter LDRs are easier to predict than longer LDRs.

## 3. Discussion

Missing backbone atoms in X-ray structures have been widely used as a proxy of IDPs/IDRs. In the case of relatively short regions, the missing electron density is often a consequence of alternative conformations in highly flexible areas, whilst for very long regions it most likely corresponds to unstructured portions of the polypeptide chain. However, X-ray diffraction is not a unique technique to identify intrinsic disorders. The recent improvements in the field of NMR have contributed to the study of IDPs/IDRs considering their conformational ensembles at atomic resolution. The advances in computational techniques jointly with NMR spectroscopy provide valuable structural and dynamic data of IDPs/IDRs, playing an important role in understanding their complex conformational behavior in the cell. These two techniques are key to studying the continuum between order and disorder in IDP ensembles [40].

In this work, we found 3133 different LDR protein sequences from missing backbone atoms in X-ray structures. The use of X-ray crystal structure in this study deserves a specific comment, since it is empirically well known that macromolecules with long flexible parts will tend to resist crystallization. It is common practice among crystallographers to produce different constructs of the same protein in order to reduce the flexible portions and, in doing so, favor crystal growth. In this sense, we would expect that our analysis underestimates the fraction of disordered regions present in the protein world. Most likely a larger fraction of disorder is present in the proteins that have not been yet crystallized. We think that our analysis extends and complements our knowledge of LDRs from missing electron density. Our previous works regarding long and short disordered regions were focused on different aspects. On one hand, we assessed disorder predictors on LDRs present in a curated resource as DisProt database. Suggesting that predictors heterogeneity can capture different protein disorder flavors and can benefit from high-quality data [10]. On the other hand, we performed an extensive analysis of ID present in the protein universe of the UniProt database, based on predictions [37].

One of the main reasons for developing computational approaches was the scarcity of experimental data to make hypotheses. Despite this, predictors have given some interesting hypotheses with respect to LDRs, such as a functional analysis in full proteomes [11] and biological processes [12]. However, although predictors have good precision and can generate large quantities of data, they still contain systematic errors. For instance, on LDR proteins predictors achieve high specificity and precision but low sensitivity. While predictors prove a performance considerably above random, nevertheless substantial errors remain and a large fraction of disorder residues remains undetected. This is also in agreement with a previous work on disorder predictors performance from X-ray missing residue data [41]. Five year later, despite more data being available in the PDB, it is still difficult to accurately predict LDRs.

Our experimental LDR set is also significantly different from the currently available curated databases DisProt and IDEAL. It is important to stress that our data are not simply PDB entries, but rather multiple X-ray experiments assigned to multi-domain UniProt sequences. Different X-ray experiments may be assigned to the same sequence with the final disorder/structure decision based either on majority evidence or complete lack of structure. This should produce a more stable definition since it will remove noise, e.g., missing residues arising from low resolution data or not well refined crystal structures. 

It has been widely studied in the field of IDPs/IDRs their capacity to adopt a folded state when interacting with another protein. Diverse mechanisms and databases have been proposed in the literature including mutual synergistic folding [42,43,44,45], fuzzy complexes [46,47], couple folding and binding [29,48], and those proteins participating in liquid-liquid phase separation [49]. This group of proteins have disordered segments in their monomeric forms which can undergo folding upon binding or that are part of a wobbly/mobile domain. In our dataset of LDRs by subtracting the “zero” to the “majority” consensus (mixtures of disorder and structure), we identified some of these regions mentioned above (ca. 10% of the “majority” consensus regions). These regions were not included in the “zero” consensus because they are ordered/structured at least in one of associated PDB structures. Predictors performance for these regions (Table S3) is even worse than “zero” and “majority” consensus, with a lower sensitivity and MCC. However, more future work is needed to better assess and analyze these regions.

## 4. Materials and Methods

### 4.1. Long Disorder Data

All UniProt [50] sequences with at least one structure in the Protein Data Bank (PDB) [18] (released until the 31st of December, 2018) were retrieved from MobiDB [51] (Dataset S1). A total of 44,090 protein entries were found, having structures coming from X-ray diffraction, Nuclear Magnetic Resonance (NMR) and/or cryo-EM. After removing NMR and cryo-EM structures, the total amount of proteins used for this analysis was 37,395, composed of 121,942 different PDB files and 314,829 protein chains. 

Structure quality metrics reported in the PDB [27] were assigned to each X-ray structure by using the recently published dataset by Brzezinski et al. [25], available at https://github.com/dabrze/pdb_structure_quality. Six key validation metrics were used to measure the overall structure quality: Resolution, R-free, clashscore, Ramachandran outliers, rotamer outliers and RSRZ outliers (normalized real-space R-factor).

The structure quality estimation for NMR and cryo-EM models is difficult to assess in order to be comparable with crystallographic structures. Additionally, the way that cryo-EM determines the resolution is different [23], and consequently their maps and models could not be equivalent [24]. For the above mentioned reasons and the small contribution of these methods to our dataset, we only focused our analysis using X-ray structures.

Disorder consensus definition has been extensively used to combine disorder information coming from different predictors and data sources [28,29,37,51,52]. Disordered residues at the UniProt protein level were assigned using two different consensus strategies, namely “majority” and “zero” rule, to combine missing residues from different PDB chains. In the “majority rule” (Dataset S2) a segment is considered a LDR if it is disordered for at least 30 residues in the majority (more than 50%) of the structures corresponding to the same polypeptide chain, i.e., mapping to the same UniProt entry. The “zero rule” (Dataset S3) is applied to the subset of majority cases where all the structures have a given LDR. Those protein fragments for which no PDB structures are available are considered as unknown/undefined. 

Long disordered regions of at least 30 residues from DisProt (version 2019_08) [28] and IDEAL (release April 2019) [29] were used for comparison. The same majority rules were applied to IDEAL, and only disordered regions annotated with “disorder” and “high_rmsd” tags were considered. 

### 4.2. GO-Terms Enrichment Analysis

Functional enrichment was calculated for the first 4 levels of the Gene Ontology (GO) [33,34] graph as available in January 2020. Fisher’s exact statistical tests were carried out for the enrichment analysis using the LDR (3133 proteins) and fully disordered proteins (34 LDR proteins with at least 70% of disorder content) as targets sets, and all UniProt [10,41] sequences with at least one PDB structure as background (40,200 proteins). A term was considered enriched if the *p*-value with Bonferroni correction was outside the 95% confidence interval of the mean (*p* < 0.05).

### 4.3. Disorder Prediction and Evaluation

The assessment approach is the same as described in a previous work also based on missing residues in the PDB [41]. Predictions were downloaded from MobiDB [51] which includes the following programs (disorder definition used in parenthesis): ESpritz (X-ray, NMR and DisProt; [39]), IUPred (short and long; [53]), DisEMBL (hot loops and remark 465; [54]), VSL2b (combination of X-ray and DisProt; [22]), GlobPlot (globularity; [55]) and MobiDB-Lite (single consensus-based prediction; [53]). A total of 10 prediction methods with different disorder flavours were evaluated on the majority and zero LDR proteins. Unknown residues on consensus definitions were excluded from the assessment. Since disorder prediction is a binary classification, performance measures as accuracy, precision, specificity and recall were calculated per residue. The following set of measures were considered: Balanced accuracy (BAC), F1 score, Matthews correlation coefficient (MCC), positive predictive value (PPV) or precision, true negative rate (TNR) or specificity and true positive rate (TPR) or recall.

## 5. Conclusions

A large dataset of diverse proteins with LDR is available to be used as a training set in disorder prediction techniques, as well as target IDPs to be included in the curated resources. Training a novel predictor on this large amount of quality data using state-of-the-art machine learning algorithms can only enhance our understanding of the phenomenon and improve their detection. Additionally, IDRs identified in this work could be used as a high-quality base ground to help in the annotation and identification of IDPs. A clearer picture will emerge as more structures are deposited each year in the PDB. Missing residues provide a valuable source of LDRs which tend to be overlooked in PDB as a data source. Moreover, we provided two different ways to combine PDB information at the protein level which can identify fold upon binding regions and showed that these regions are more difficult to predict and are largely under-detected by disorder predictors. In this work we demonstrate that PDB is not only the main repository of macromolecular structures but is also a good source to explore the (un)structure—function paradigm looking at disorder regions exposed to different experimental conditions, proving that most of the LDRs found have a biological role. 

## Figures and Tables

**Table 1:** Dataset composition. The number of proteins, short and long disordered regions, residues, and median protein length is shown for proteins with long disordered regions (LDR, region length 30+), short disordered regions (SDR, region length 5+ and <30) and structured proteins without missing residues or with disordered regions shorter than 5 residues. Disordered regions are defined using the majority rule. Residues are unknown if they are not covered by any PDB structure in the corresponding UniProt entry. More than one disordered region per protein may be present. Percentages are rounded and may sum up to more than 100.

Dataset	Proteins	Median Protein Length	Short Disordered Regions (SDR)	Long Disordered Regions (LDR)	Missing Residues (Disordered)	Observed Residues (Structure)	Unknown Residues
Proteins with LDRs	3133	473.0	3742	3553	270,656 (14.1%)	1,140,513 (59.4%)	509,174 (26.5%)
Proteins with SDRs	15,968	358.5	25,087	0	303,611 (3.9%)	4,724,722 (60.4%)	2,787,457 (35.7%)
Structured proteins	18,294	274.0	0	0	28,338 (0.4%)	4,242,966 (62.8%)	2,485,372 (36.8%)
Total	37,395	441.0	28,829	3533	602,605 (3.7%)	10,108,201 (61,3%)	5,782,003 (35.1%)

**Table 2:** Disorder prediction evaluation on proteins with long disordered regions (LDRs) using the majority consensus. Methods are ordered by Matthews Correlation Coefficient (MCC). In bold the best value and underlined the second best for each measure.

	MCC	F1 Score	Accuracy	Precision	Specificity	Recall
**Espritz-X**	**0.456**	0.498	0.672	0.738	0.968	0.376
**IUPred-short**	0.411	0.473	0.662	0.656	0.954	0.370
**MobiDB-Lite**	0.389	0.350	0.606	**0.872**	**0.992**	0.219
**VSL2b**	0.384	**0.516**	**0.720**	0.432	0.800	**0.640**
**IUPred-long**	0.375	0.456	0.655	0.590	0.939	0.372
**DisEMBL-465**	0.364	0.416	0.633	0.644	0.960	0.307
**Espritz-N**	0.361	0.485	0.683	0.478	0.872	0.493
**Espritz-D**	0.257	0.214	0.558	0.746	0.990	0.125
**DisEMBL-HL**	0.209	0.388	0.622	0.316	0.742	0.503
**GlobPlot**	0.192	0.329	0.588	0.368	0.879	0.297

## Supplementary Materials

The following are available online at https://www.mdpi.com/1422-0067/21/12/4496/s1.

## Abbreviations

IDR	Intrinsically disordered region
IDP	Intrinsically disordered protein
LDR	Long disordered region
SDR	Short disordered region
