# Supplementary material for: Experimentally Determined Long Intrinsically Disordered Protein Regions Are Now Abundant in the Protein Data Bank
Source: Int J Mol Sci. 2020 Jun 24;21(12):4496. doi: 10.3390/ijms21124496 (PMC7349999; doi:10.3390/ijms21124496)
Supplement: Supplementary file 1 [file ijms-21-04496-s001.zip › ijms-830071 final suppl 1/Monzon_supplementary_files/Monzon_et_al_Supl_material_IJMS.pdf]

Experimentally determined long intrinsically disordered protein regions are now abundant in the Protein Data Bank

Alexander Miguel Monzon[1,*], Marco Necci[1,*], Federica Quaglia[1], Ian Walsh[2], Giuseppe Zanotti[1], Damiano Piovesan[1,°] and Silvio C. E. Tosatto[1,°]

[1]Department of Biomedical Sciences, University of Padua, Padua, Italy
[2]Bioprocessing Technology Institute, A*STAR, Singapore
[*]These authors contributed equally to this work
°Co-corresponding authors

°Corresponding authors: damiano.piovesan@unipd.it and silvio.tosatto@unipd.it

Supplementary Material

Supplementary Figures

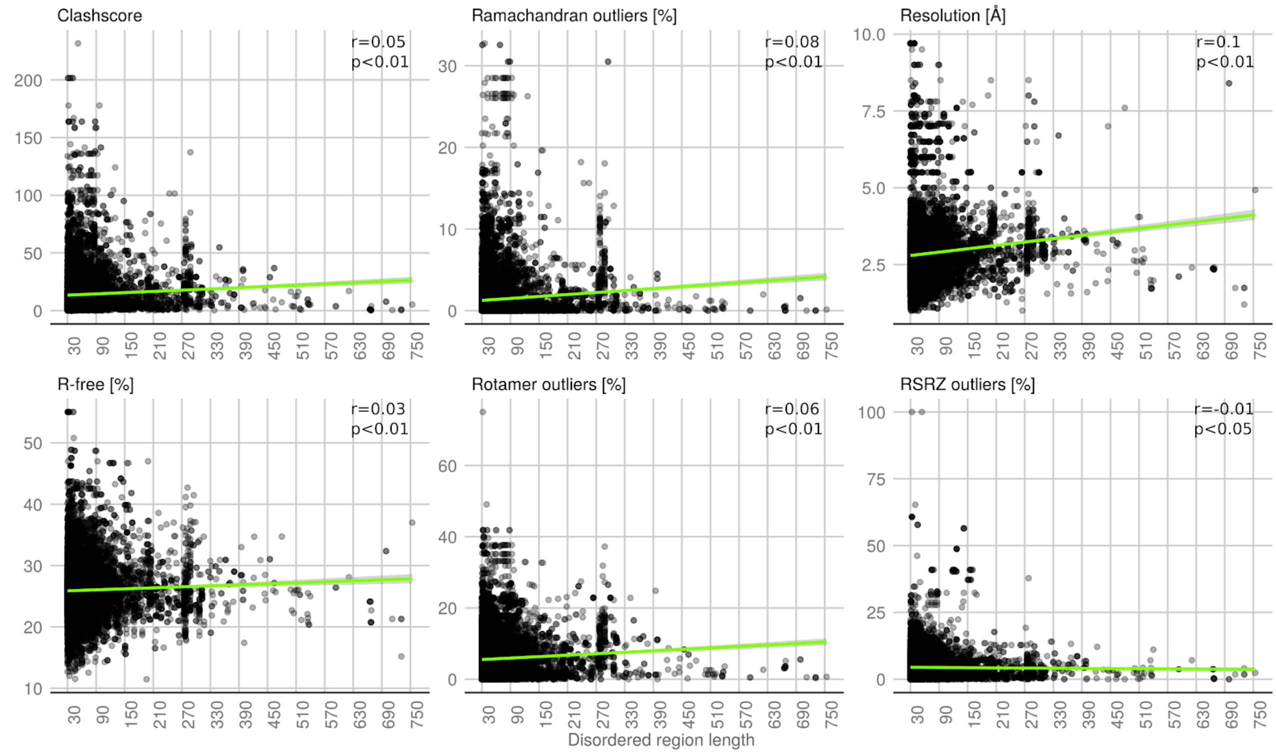

Figure S1: Scatter plots of different structure quality metrics and disordered region length. On the X-axis the disordered region length, calculated for each PDB chain with LDRs (at least 30 residues length). On Y-axes the different quality metrics corresponding specified on the title of each subplot. Pearson's correlation coefficients (r) and P-values (p) are shown for each subplot.

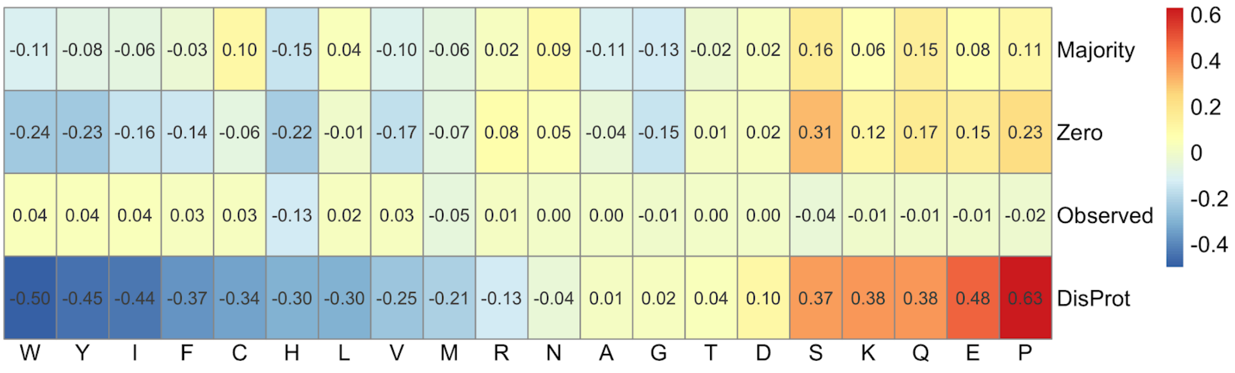

Figure S2: Heatmap of amino acid composition enrichment. Each cell represents the fold increase (red) or decrease (blue) compared to the PDB SEQRES amino acid distribution. Majority and Zero represent the LDRs amino acid frequency distribution for each consensus rule. Observed is the amino acid frequency distribution of all structured residues in the PDB. The amino acids are ordered by DisProt enrichment values (baseline composition distribution of disordered proteins).

Supplementary Tables

UniprotID	Region start position	Region end position	Final Decision	DisProt accession (release: 2020_05)
O60494	1389	3623	NO MENTION	-
Q9BE39	807	1935	NO MENTION	DP02522
O60494	121	931	NO MENTION	-
B2HN69	1	731	NO MENTION	-
G0S3L5	1	699	DISORDERED	DP02520
E5XP76	1	667	NO MENTION	-
P20676	332	973	DISORDERED	DP01075
Q6RKB1	640	1174	NO MENTION	-
A5HC98	1746	2263	DISORDERED	DP02521
Q08345	1	505	NO MENTION	-
Q01101	10	510	NO MENTION	DP01023
P46674	806	1301	DISORDERED	DP02481
O60563	262	726	NO MENTION	-
P35637	60	507	DISORDERED	DP01102
Q8ZRP0	1	422	DISORDERED	DP02483
Q5A3P6	524	944	DISORDERED	DP02500
P32504	538	956	DISORDERED	DP02033
A5YV76	2115	2512	DISORDERED	DP01026
Q9TY14	1	378	DISORDERED	DP00749
A0A0J9X1Q5	1	371	DISORDERED	DP00913
Q86U44	1	367	NO MENTION	-
P02671	220	581	DISORDERED	DP02130
Q9NYB9	156	513	DISORDERED	DP02386
Q01970	883	1234	DISORDERED	DP02477
P0DOC6	1	349	NO MENTION	-
Q9BIM8	1	342	DISORDERED	DP02877
Q13740	246	583	DISORDERED	DP02515
O75533	1	333	DISORDERED	DP01863
P08240	1	331	DISORDERED	DP00893
P20676	1	326	DISORDERED	DP01075
Q13888	1	325	NO MENTION	-
Q5HLM5	1	312	NO MENTION	-
Q92558	185	494	DISORDERED	DP02529

Q9UKL0	4	310	DISORDERED	DP02523
Q01080	116	415	DISORDERED	DP02519
P18564	492	788	DISORDERED	DP02530
C4R4Y0	1454	1743	DISORDERED	DP02526
P22473	1	288	DISORDERED	DP02487
P12537	304	585	DISORDERED	DP02508
Q84852	17	298	DISORDERED	DP02507
P04050	1456	1623	DISORDERED	DP02527
Q80UG2	952	1229	DISORDERED	DP02528
P15825	74	348	DISORDERED	DP02536
P14448	237	505	DISORDERED	DP00233
Q86UE8	191	455	DISORDERED	DP02475
O53168	1	264	DISORDERED	DP02505
Q676U5	49	307	DISORDERED	DP02148
P11961	170	428	NO MENTION	-
P08621	181	437	DISORDERED	DP02171
P54652	386	639	NO MENTION	-
P09327	360	613	DISORDERED	DP02510
P46674	1	252	DISORDERED	DP02481
P35828	1	248	DISORDERED	DP02504
Q28146	31	277	DISORDERED	DP02503
Q64487-12	1019	1265	DISORDERED	DP02517
E6YFW2	313	558	NO MENTION	-
Q05022	1163	1407	DISORDERED	DP02513
P0AD27	1	245	NO MENTION	DP02484
P40709	1	242	NO MENTION	DP02485
A0R1T8	1	239	DISORDERED	DP02502
A5YKK6	1605	1841	DISORDERED	DP02524
P56287	444	678	DISORDERED	DP02516
Q06696	162	395	DISORDERED	DP01611
Q8A6W3	1	234	DISORDERED	DP02506
P00800	1	232	DISORDERED	DP02501
Q5A3P6	1	232	DISORDERED	DP02500
P36106	1	231	DISORDERED	DP02476
A0KJC7	1	230	DISORDERED	DP02499
P36106	366	594	DISORDERED	DP02476
P50616	117	345	DISORDERED	DP00794
S6B291	238	465	NO MENTION	-
A7YK37	1	227	DISORDERED	DP02498
P36594	1509	1735	DISORDERED	DP02514

P05844	735	960	DISORDERED	DP02497
Q8ZRW0	1	225	DISORDERED	DP02518
O60502	696	916	DISORDERED	DP02479
O70038	116	336	NO MENTION	-
P56926	633	852	DISORDERED	DP02496
P15311	298	515	DISORDERED	DP00775
Q928V6	133	349	DISORDERED	DP02495
Q9BJX6	1	217	DISORDERED	DP00800
O56139	1	216	DISORDERED	DP02494
P21401	154	369	DISORDERED	DP02493
Q808Y3	1	216	DISORDERED	DP02492
Q9WBP8	1	216	DISORDERED	DP02491
Q9Z4P9	375	589	NO MENTION	-
D1A4G7	200	413	DISORDERED	DP02490
Q9P2K3	1	214	DISORDERED	(isoform 3) DP02408
B4Y891	1	213	DISORDERED	DP01984
Q9YIJ1	1	208	DISORDERED	DP02489
O28769	30	236	DISORDERED	DP02488
P9WGI1	1	206	DISORDERED	DP02525
P22473	301	506	DISORDERED	DP02487
H0W0T5	362	565	DISORDERED	DP00870
Q12102	423	625	DISORDERED	DP02480
A7ZUK2	932	1134	DISORDERED	DP02486
P0A8T7	932	1134	NO MENTION	-
Q8NCM8	1054	1254	NO MENTION	-
Q6SJQ7	138	337	DISORDERED	DP02478

Table S1: Manually curated longest LDR. Uniprot accession number, region start and end positions, final curator decision and DisProt accession code. No mention means that no evidence was found to relate the presence of missing residues with intrinsic disorder.

	MCC	F1 score	Accuracy	Precision	Specificity	Recall
**Espritz-X**	**0.461**	**0.508**	0.679	0.717	**0.965**	0.393
**VSL2b**	0.413	0.479	0.668	**0.635**	0.95	**0.385**
**Espritz-N**	0.399	0.368	0.613	**0.852**	**0.991**	0.235
**IUPred-short**	0.379	0.506	**0.722**	0.414	0.794	**0.65**
**IUPred-long**	0.379	0.463	0.662	0.571	0.935	0.389
**DisEMBL-465**	0.363	0.419	0.636	0.621	0.957	0.316
**DisEMBL-HL**	0.361	0.483	0.687	0.461	0.867	0.507
**MobiDB-Lite**	0.266	**0.225**	**0.561**	0.738	0.989	0.133
**GlobPlot**	0.203	0.377	0.621	0.301	0.738	0.503
**Espritz-D**	0.193	0.329	0.591	0.356	0.877	0.305

Table S2: Disorder prediction evaluation on LDR proteins using the "zero" consensus. Methods are sorted based on MCC. In bold the best value and underlined the second best for each measure.

	MCC	F1 score	Accuracy	Precision	Specificity	Recall
**Espritz-X**	**0.391**	0.428	0.643	0.653	0.968	0.318
**IUPred-short**	0.363	0.418	0.641	0.587	0.957	0.324
**VSL2b**	0.34	**0.458**	**0.703**	0.37	0.806	**0.6**
**DisEMBL-465**	0.328	0.379	0.622	0.565	0.959	0.285
**MobiDB-Lite**	0.325	0.269	0.577	**0.823**	**0.993**	0.161
**IUPred-long**	0.312	0.378	0.623	0.52	0.948	0.297
**Espritz-N**	0.296	0.412	0.653	0.397	0.877	0.429
**Espritz-D**	0.212	0.177	0.546	0.636	0.989	0.103
**DisEMBL-HL**	0.207	0.359	0.63	0.274	0.74	0.519
**GlobPlot**	0.147	0.272	0.569	0.295	0.886	0.252

Table S3: Disorder prediction evaluation on LDR proteins subset ("majority" - "zero" consensus). Methods are sorted based on MCC. In bold the best value and underlined the second best for each measure.

Supplementary Data

Dataset S1: Semicolon separated file with all missing residue regions for all PDB and chains in the dataset.

Dataset S2: Semicolon separated file with all "majority" consensus regions in the dataset.

Dataset S3: Semicolon separated file with all "zero" consensus regions in the dataset.
